# Supplementary material for: COVID-19 pandemic preparedness period through healthcare workers’ eyes: A qualitative study from a Romanian healthcare facility
Source: PLoS One. 2021 Oct 6;16(10):e0257381. doi: 10.1371/journal.pone.0257381 (PMC8494332; doi:10.1371/journal.pone.0257381)
Supplement: Attachment — Submitted filename: rev_2_8.8.2021.pdf [file pone.0257381.s004.pdf]

8.8.2021

Dear Editor,

Authors covered majority of my recommendations. I thank to them. However, there are still lacking points which have been emphasized below:

1. I could not see the “overall” recommendations as a separate part of the manuscript.
2. The authors stated that their study was the first study. However, there may be similar studies and the authors could not have accessed. They should change their sentence.
3. It is still not clear how the authors have connected the “Bridge to heaven” part with evidence-based medicine.
4. As it is a qualitative research, social scientist’s evaluations should be officially received before giving the decision.

Note. No need to send the revised manuscript to me, Editorial Office can give the final decision especially using the social scientist's decision.
