# Supplementary material for: COVID-19 pandemic preparedness period through healthcare workers’ eyes: A qualitative study from a Romanian healthcare facility
Source: PLoS One. 2021 Oct 6;16(10):e0257381. doi: 10.1371/journal.pone.0257381 (PMC8494332; doi:10.1371/journal.pone.0257381)
Supplement: Attachment — Submitted filename: Response to Reviewers.docx [file pone.0257381.s001.docx]

Response to editor’s and reviewers’ comments:

We were happy to have the opinions and comments of the reviewers and an editor. We are sure that their comments made our article better, and we hope we succeeded in making the desired changes. Thank you for your time and effort!

Journal Requirements:

When submitting your revision, we need you to address these additional requirements.

Please ensure that your manuscript meets PLOS ONE's style requirements, including those for file naming.

We revised the PLOS ONE’s style requirements, including those for file naming.

 The title of the submission should specify that the research is representative of a Romanian healthcare facility.

We added in the title that the study is representative of a Romanian healthcare facility.

We note that you have indicated that data from this study are available upon request. PLOS only allows data to be available upon request if there are legal or ethical restrictions on sharing data publicly.

The informed consent stipulated that the transcripts will be shared only with the team members. Therefore, the Colentina Hospital Ethics Committee of Research considers that we do not have the right to share the transcripts with you. If providing you the transcripts is mandatory, in order to fulfil your request, we should obtain an additional informed consent from each and every participant. Should you need more information, please contact the Colentina Hospital Ethics Committee of Research, at Colentina Hospital, Soseaua Stefan cel Mare 19-21, sector 2, 020125 Bucharest, Romania. (President of the Colentina Hospital Ethics Committee of Research: Gheorghe Andrei Dan, [andrei.dan@gadan.ro](mailto:andrei.dan@gadan.ro))

Reviewer #1: Thank you for the opportunity to review your manuscript. The topic is important and will continue to be so for a long time.

It would have been useful to explain for international readers what you mean by 'the preparedness period of COVID-19.

We added the following explanation in the Introduction section “. The preparedness period is represented by the phase when there is no direct contact with the threat, but all efforts are directed towards anticipating the course of events, preparing responses to those events and trying to translate those responses into practice”.

Your research question was clearly stated. However, whilst your aim was to consider perceived weaknesses and strengths, the latter was missing from your analysis and discussion.

Yes, this was our aim. But participants were overwhelmed by negative feelings, so they did not provide us enough information regarding the strengths perceived by them, at least not enough information in order to generate a code or a theme. We added this in the results section.

Your approach to thematic analysis was clear and use of Table 3 illustrated the themes and subthemes. Quotes were used to illustrate the themes. You stated that saturation of data occured after 11 interviews (line 112) and providing some further explannation of this would be useful. In addition, if saturation occured after 11 interviews what was the purpose of conducting six more interviews?

We performed more than one interview per day and we did not manage to transcribe every interview immediately after recording it. Therefore, we had already taken the last six interviews when we realized that we had already reached data saturation. Therefore, as a gratitude gesture for the time offered by our colleagues, and their involvement in our project, we decided to include them in the study.

Did your analysis of the residents interviews highlight more negative experiences as you suggest this might be the case in your introduction.

Yes, there were some differences regarding residents’ emotions and experiences, when compared with those of the seniors. We are aware that including residents in our study may have influenced the codes and the themes, but our aim was not to analyze their experiences by comparison with seniors’ experiences, but rather to ascertain an overview of the HCWs emotions and experiences.

I note that on line 314 you refer to bullying, and this is quite a strong statement, but no quotes are provided to support this.

Bullying was not present *expressis verbis* in our transcripts; it transpired indirectly during the interviews. We also reanalyzed the transcripts; reading them months after writing them, and after dealing with Covid-19, we realized that this was, as you said, a strong statement, which might have been influenced by our emotional status at the moment. Therefore, we decided to delete that part from the results section.

In your conclusion (line 417) you state that further attention should be paid to factors that may help HCWs regain their balance, but what does that mean?

We rephrased that paragraph as it follows: “Identifying sources of support during this period, by asking HCWs what they needed , what helped them or what would have helped them, and not by searching for a feedback in regard with alleged forms of reinforcement, should be further assessed in longitudinal qualitative studies.”

I did wonder whether some discussion of 'uncertainty' and also 'psychological safety' could have added more in depth understanding to your discussion.
An additional paper that might be helpful to you is the following;
Shanafelt, Et. et al (2020) Understanding and addressing sources of anxiety among health care professionals during the COVID-19 pandemic. Journal of the American Medical Association April7th.

We thank you for your recommendation; we read the paper, and we further detailed the discussion section.

Reviewer #2: This is a narrative or a commentary rather than a piece of scientific research. The authors have presented a series of responses to a standard set of interview questions in a journalistic format with no solid conclusion drawn. No information was provided about the number of eligible subjects and the response rate. Participants were voluntary and therefore selection bias is inevitable. With a sample size so tiny and such skewed demographics, it is impossible to draw any conclusions whatsoever.

In qualitative research, in order to identify themes and express results, the authors immerse into data; therefore, we consider that a narrative or a commentary component, as you have stated, which is also subjective, is inherent to any qualitative study, by its own nature. Neither COREQ nor SRQR guidelines for reporting qualitative research stipulate to provide information about the number of eligible subjects and the response rate; as far as we know, these requests are necessary when reporting results of surveys, in quantitative research. Participants had to be voluntary, because nobody can constrain someone to enroll in a study, nor to offer an interview. We understand that it may have been a selection bias, but we believe that a certain degree of selection bias is present in every qualitative study. Regarding the sample size, some scientific papers say that saturation appears after twelve interviews. We cannot say that this is universally true, but is certain that qualitative research tends to use smaller samples size than quantitative research. Besides, according to the scientific literature, sample size is in close connection to the concept of saturation, and remains a widely used rationale for sample size in qualitative research; therefore, when saturation occurs, the sample size is enough, no matter the number of participants enrolled in the study. In our case, saturation occurred after 11 interviews, therefore 11 participants would have been enough. In regard with demographics, qualitative researchers may aim to sample for diversity of perspectives, or for typicality or homogeneity of perspectives; as far as we know, sampling for heterogeneity (in our case, age heterogeneity), is not a mistake, nor a weak point in a qualitative study. Our aim was not to identify patterns through various age categories, nor to compare them, but to have an overview of the health care workers emotions and experiences through this difficult time.
