# Supplementary material for: COVID-19 pandemic preparedness period through healthcare workers’ eyes: A qualitative study from a Romanian healthcare facility
Source: PLoS One. 2021 Oct 6;16(10):e0257381. doi: 10.1371/journal.pone.0257381 (PMC8494332; doi:10.1371/journal.pone.0257381)
Supplement: Attachment — Submitted filename: Response to reviewers.docx [file pone.0257381.s002.docx]

Reviewer #3:

First of all, we want to thank the third reviewer, for her important commentaries, and her guidance.

1. First, I want to comment on Reviewer 2's critiques, which I found to be laughable. Thematic analysis is science and is not 'journalism.' It doesn't take a genius to know there are methods beyond run of the mill positivism. Google it.

   More specific to this paper, I do believe the study has potential, but there are some issues that need to be addressed prior to publication. I point these out by line number below.

   2. 62-why is the 'preparedness period; the hardest to endure? I would think it would be the period in which the disaster - that being the actual instance of pandemic would be the most difficult. Please clarify.

We believe that the preparedness period is the hardest to endure, much harder than the actual pandemic phase, because during preparedness you are, at best, constantly exposed to all sorts of news and information from the experience of others, and since you are not involved “in the fight”, you are only left with analyzing and overthinking all those information, which is not always productive, but rather it represents a huge source of anxiety. At worst, you know nothing about the disease, the rate of transmission, the mortality, the actual effectiveness of the personal protective equipment, and one can only estimate all those things; during the time you wait for an emerging crisis to reach, some of us, if not most of us, tend to overestimate all those “magnitude parameters”, which is also a big source of anxiety. Our experience, and also our colleagues experience, confirmed our feelings, and once we were involved in the process of taking care of COVID-19 patients, we focused on doing our best on helping these people, rather than thinking at everything that was happening or would happen, and so the anxiety from the preparedness period diminished.

3. 70-'weakness and strengths' of what specifically?

We aimed to ascertain the perceived weaknesses and strengths regarding the way in which the Healthcare system and the Healthcare units were organizing and preparing for the pandemic response; consequently, those perceived weaknesses and strengths would reflect in the healthcare workers well-being. We added this clarification in the Materials and Methods section. After our first revision, in accordance with the first reviewer commentaries, we added in the Results section the fact that the participants were overwhelmed by negative feelings, so they did not provide us enough information regarding the strengths perceived by them, at least not enough information in order to generate a code or a theme.

4. 71-please describe why it is a 'mandatory process' and what 'errors' you are talking about - are they those mentioned in the section above? If so, please state explicitly so the reader doesn't get off track or confused like I did. Also, why was doing this in Europe, specifically in Romania, important?

Yes, the errors we were referring to, are represented by the weaknesses that they perceived; we added this in the paragraph, in order to clarify what we meant. We believe that this is a mandatory process, because only by analyzing the experience of the healthcare workers during pandemic time, we can understand what issues need to be addressed in the future, in order to improve the healthcare system response to a pandemic. Even though we have dealt with previous outbreaks, none of them had this magnitude, and clearly anticipating and planning a pandemic response in “neutral” times is not enough, which is reflected in the lack of personal protective equipment supplies, the lack of trainings regarding donning and doffing, and so on. Moreover, there are major differences between countries, and continents, regarding culture, habits, way of socializing, and clearly regarding the quality of the healthcare system. We believe that it is important to ascertain perceived weaknesses and strengths from various people and countries, in order to have a proper larger frame over the response to a pandemic, which will permit us a better understanding of the experience, and consequently, as we hope, it will lead to implementing some measures and improving future pandemic responses.

5. 73-COREQ Criteria - What is that? Please define and describe for unfamiliar readers. Also, why did you choose a qual study? I understand you mention there were no prior qual studies, but this in itself doesn't mean there should be. The need for such research is driven by the current state of the work and gaps left in them - what is the specific knowledge gap this fills? Why do your question(s) necessitate a qual approach? The method is driven by the questions and the need to answer them/it. So, tell the reader why this work was crucial.

COREQ criteria is the abbreviation for “Consolidated criteria for reporting qualitative research”, which represents one of the two reporting guidelines regarding qualitative research, suggested by the EQUATOR (Enhancing the QUAlity and Transparency of Health Research) Network, in order to improve the reliability and value of published health research literature; we added what COREQ stands for in the Materials and Methods section.

As we stated in the introduction, at the moment we performed, and wrote our study, there were two qualitative studies developed in China, one which enrolled nurses, while the other one involved nurses and doctors. As we mentioned above, there are major differences between China and other countries from all over the world, starting with the politic regime, and finishing with the healthcare system, and culture, which have a major impact in regard to how people respond to restrictive measures, dissemination of information, pandemic response and how people perceive all those facts. Taking all those mentioned above (and also those mentioned in our answer to the previous question), we believe that it is important to ascertain more points of view, in order to have a larger frame over how healthcare workers perceived the pandemic preparedness period (and for future studies how they perceived the pandemic response), in order to improve, on a global level, future responses. Besides, as we stated in the discussion section, our study is the first one to include residents, besides doctors and nurses, which, even though is inherent to a more heterogeneous information, and for some people, this may represent a minus, we consider that it is necessary in order to have a full view over the perceived weaknesses, moreover because residents were an important part in the response to the pandemic, at least in our country. We consider that a survey could not provide an in-depth understanding of the healthcare workers perceptions and feelings, maybe not even one with an endless number of questions (which would, of course, be unrealistic); we believe that qualitative research is the proper method to use when it comes to in-depth understanding and analyzing people emotions and feelings regarding sensitive issues.

6. 79 - What do you mean by 'rich' data? Why is it important?

By rich data we mean detailed interviews, which offer information that permit in-depth understanding of the experience and proper analysis. Otherwise, there is not much difference between a qualitative study and a survey performed online.

7. 80- What sort of validity are you trying to establish because it is different given your methods. Maybe you are talking about trustworthiness? It might be helpful to refer to some seminal texts here (I know some specific methods texts are talked about later). Glaser, Strauss, and Corbin and their variety of works might be useful in this section and will help fend off the qual hater trolls that always come abut try to wreak shenanigans on these studies.

By validity, we meant that we wanted to evaluate if the questions included in the interview would be able to generate the data necessary for investigating how HCWs perceived the preparedness period. We rephrased that sentence in order to be clearer. We ensured trustworthiness by performing member checking.

8. 107-What do you mean by "was realized?"

We meant that the person which conducted the interviews was prepared for this task by performing an extensive documentation.

My next comments center more on the themes and findings of the work. I am going to point towards lines 167-169 as to what I see as the most interesting contribution in what is a somewhat messy bunch of data which indicates the analysis isn't quite yet done. The point raised in those lines (and of course, I have not seen or analysed the actual data) indicates that a breeching experiment (a la Garfinkle) has indeed been performed to some extend via the pandemic. What this accomplished is an upending of reality and the illusion of control that not only medicine has over pathogens, but also humans have over death (and by extension medicine has over death). So this is much more than 'shades of grey' - which you might reconsider the title given that wretched book and movie - COVID has torn apart those illusions of control and people, including the HCW you studied and are a member of, have been thrust into.

We are not quite sure if you understood that we used in our interviews Garfinkle’s research method, known as “breaching experiment”; if this is the case, we are sorry to inform you that we did not do this. We renamed the first theme, in order to avoid any form of association with that book, even an unwanted one.

You might want to read the following paper: <https://www.academia.edu/20128995/Disaster_Preparedness_as_Social_Control> - it is a different case but the methods and themes are related.

I think this larger frame might help make sense of all this stuff you have going on here, which as it stands is a lot to digest. I do believe more analysis is necessary even if saturation was reached (totally possible at your # of interviews, no question), themes should be condensed, and the way you describe your data within themes needs more contextualization - you just kind of throw quotes there and don't really massage them into findings and conclusions for the reader - you must do this for it to make sense. If so, you will have a really great, publishable paper. I know it is somewhat discouraging to go back to analysis, but I speak from experience when I say, I have done this numerous times and it has only made my work that much better.

We read with great interest the paper indicated by you, and we made some major changes in the Results section.
