# Supplementary material for: COVID-19 pandemic preparedness period through healthcare workers’ eyes: A qualitative study from a Romanian healthcare facility
Source: PLoS One. 2021 Oct 6;16(10):e0257381. doi: 10.1371/journal.pone.0257381 (PMC8494332; doi:10.1371/journal.pone.0257381)
Supplement: Attachment — Submitted filename: Response to Reviewers.docx [file pone.0257381.s003.docx]

Dear Ms Frey,

Please find attached our responses to the third and fourth reviewers. We hope that you will find this final revision in accordance to the reviewers’ requests.

Reviewer #3:

1. To clarify, I hope the authors understand this was a comment directed towards the prior reviewer and not the authors themselves.
2. I understand the authors points in response to my question and they are believable, especially since the authors define it as such:

   "The preparedness period is represented by the phase when there is no direct contact with the threat, but all efforts are directed towards anticipating the course of events, preparing responses to those events and trying to translate those responses into practice. We believe that this period, which is characterized by a state of expectation, is one of the hardest to endure."

   I am left with a couple important questions - 1) are the authors drawing on their own experiences too as they describe in their response letter? If so, then I suspect they have also engaged in a co-autoethnographic project, which adds even more legitimacy to this work (although is an often marginalized method). 2) how do they distinguish between the 'preparedness period' and the 'response period'? Perhaps, as authorities themselves of this context, these lines are blurry. Which in itself is extremely important as a finding and it worthy of mention. I am not trying to push more literature on to the authors or more work. However, I think 2 other papers that can help strengthen both the methods and findings, could be considered:

   [https://www.academia.edu/41122099/Hurricane_Harvey_Unstrapped_Experiencing_adaptive_tensions_on_the_edge_of_chaos](about:blank)

   [https://www.academia.edu/38022452/For_a_short_time_we_were_the_best_versions_of_ourselves_Hurricane_Harvey_and_the_Ideal_of_Community](about:blank)

   The above works address the co-autoethnographic method, which as I read your response I feel is what you actually might have done aside from the content/thematic analysis - which would fit as the analytical tool that you used to approach your data.

*Thank you for your comments and for your suggestion. I believe that the following phrase from our previous response to your commentaries may have been misleading „ Our experience, and also our colleagues’ experience, confirmed our feelings”. We read the papers which you recommended to us, and we have to be honest and to admit that we were not aware of this method. We are a team of doctors in internal medicine, and we are not familiarised with all the methods in qualitative research. We practice qualitative research out of passion, because we consider that in the medical field quantitative research is not enough, and that it must be associated with qualitative research in order to have an overview image, since medicine does not consists only in numbers, associations, causality, and p values, but it also involves humanism, which, unfortunately, in the era of randomised trials and big pharma, it had been overlooked. If it was to paraphrase Kahlil Gibran, we could say „In quantitative research or in qualitative research, the two side by side raise hands together to find what one cannot reach alone”. Our aim was not to test our feelings and assumptions through this study, but to discover how did our colleagues feel and how they experienced those rough moments. So, from a moral point of view, it would not be faire to say that we performed co-autoetnography since we were not even aware of this method. Moreover, the only moment when we let our experience infer in the results, was when we refered to the importance of keep working in the same team, since this issue was adressed during the interviews by a minority of participants, and we decided to include it in the results, due to the fact that we considered this important (which we mentioned in Statements section). Regarding the difference betweent the preparedness period and the response period, as we stated in the manuscript, the preparedness period is represented by the phase when there is no direct contact with the threat,* *while the response period would imply evaluating the experience and emotions of the healthcare staff while taking care of patients with COVID-19.*

Small point - on line 247 you still refer to "shades of grey" which is fine, but you might want to determine whether or not you want to keep it based on consistency.

*We rephrased that sentence.*

Other comments regarding the response and revisions -

I wasn't suggesting you did or should perform a breaching experiment. Disaster scholars have long written on how these events (and a pandemic is very much a disaster) are akin to a breaching experiment en masse considering they reveal underlying social conditions and norms that left hidden in everyday practice. In the undoing of everyday life, disasters reveal what is left uncovered in everyday routines. Which you speak to in the conclusions. There is other important work by Martha Feldman with respect to routines as a source of change (2000) or other scholarship on the need to reproduce a sense of routine in times of disruption (e.g. Anthony Giddens and his concept of Ontological Security as written in the Constitution of Society (1991). This becomes especially important in your discussions about death, which I continue to think is fascinating.

You are almost there. I am pushing you because I really believe your work is important, especially in the downpour of COVID-related material that is just noise. This is especially true because it seems the author's major fields are medical and they are really bridging multiple disciplinary fields of sociology, anthropology, organizational studies, philosophy, in addition to methods, which is absolutely essential to deepening our understanding of how to deal with this pandemic in a more humanistic way.

Reviewer #4: Dear authors,
I have sent all of my detailed recommendations to the Editoral Office in order to contribute to the improvement of the manuscript.
Regards,

Please kindly find my recommendations for the manuscript below:

1.     The content of the manuscript may have the potential to clarify the gray zones in pandemic due to the qualitative feature of the study.

*We thank you for your appreciations.*

2.     I had the sense that the manuscript is not limited to the preparedness phase as the content covers the actual and the active pandemic phases as well. The content should be revised by making it compatible with the aims of the manuscript.

*We consider that the analysis presented in the results section is limited to the preparedness phase. Due to the fact that the virus spread to Romania later, weeks not only after the emergence of the virus, but also after the key moments when other countries were already engaged in an active fight against the virus, there are a number of references to particular situations experienced in other countries (for example the lack of personal protective equipments), which may give the feeling that the active pandemic phase is covered, but at the point when we performed the interviews, our hospital was preparing to become a COVID-19 unit, and none of the doctors have dealt with COVID-19 patients at that moment. As we stated in the introduction, the preparedness period „is represented by the phase when there is no direct contact with the threat, but all efforts are directed towards anticipating the course of events, preparing responses to those events and trying to translate those responses into practice”, and this particular issue is adressed in the results section.*

3.     The authors are recommended to use another phrase instead of “bridge to heaven” as the subthemes does not fit with the title. In addition, it is not “death”, it is “life” which the health staff aims from the beginning to the end. As a last point, the authors are recommended to use a more objective description for the themes.

*We believe that you have received the original version, which we submitted at first. This is the third time we are revising the manuscript, therefore major changes have been made until now, which we hope that will fulfill your requests. The themes have been modified previously according to the other three reviewers requests, and now the fourth theme „A bridge to heaven” does not have any subtheme. We believe that the title of the fourth theme is in accordance to its content; the theme ilustrates the fact that doctors had to hold on to what was familiar to them in order to be able to keep on track, and gives details about which were the particular things to help them achieve that. As we said, all the themes suffered major modifications, and we hope that you will find the actual content suitable. As you said, the aim of the healthcare personnel was life, but fear of death was present, an this was an important emotion which was heavily experienced at that point; so, it was mandatory to adress it in our results. Moreover, the third reviewer, which is an expert in social sciences, is particularly fond of this topic, and, as she said, considers it „fascinating”. Therefore, we hope that you will not be bothered by the fact that we have decided to keep the discussions about death. Regarding the last point you have mentioned, we tried to expose the results in an objective manner (which is more evident after the modifications we have made after the previous two major revisions); however, in our opinion, and according to Clarke and Brown, a certain degree of subjectivism is inherent to any qualitative research, and it differentiates it from quantitative research.*

4.     The authors are recommended to add current/updated literature in their discussion section as there may be recent published studies.

*We looked for recent qualitative studies to adress in the discussions section, regarding the way in which the healthcare staff experienced the preparedness period, but we did not identify any new studies regarding this topic.*

5.     Limitations should be given in a separate and a more detailed content in the discussion part. The limitation of the study type should be emphasized more strictly.

*We created a new section for Limitations, in which we further detailed what we consider to be limitations for our study.*

6.     I could not understand how the authors will use their findings to improve the system. I suggest the authors to add detailed content especially into the discussion section.

*We added in the Results section what could and should be done in order to improve the system, based on the results of our study. Unfortunately, it is not in our power to improve the system. Our aim was to gain a deeper understanding of how the healthcare staff experienced the preparedness period, which were the strengths and weaknesses in their vision, and to express them in order to gain awareness in this area. Ideas produce actions, and actions produce outcomes; therefore, after that, it is the duty of the healthcare system leaders (from macro to mycro) to implement measures like trainings for crisis situations, in order to achieve a level of preparedness that would permit doctors to face future pandemics without experiencing so acutely the fear of unknown, and the fear of death.*

7.     Recommendations are suggested to be detailed.

*It is not clear to what recommendations you are refering to; we suppose that you refer to the recommendations for improving the system, which we adressed in the previous section.*

8.     The manuscript is recommended to be evaluated by a social scientist before giving the final decision.

*The third reviewer that evaluated our paper is an expert in social sciences.*
