# Supplementary material for: COVID-19 pandemic preparedness period through healthcare workers’ eyes: A qualitative study from a Romanian healthcare facility
Source: PLoS One. 2021 Oct 6;16(10):e0257381. doi: 10.1371/journal.pone.0257381 (PMC8494332; doi:10.1371/journal.pone.0257381)
Supplement: Attachment — Submitted filename: Response to Reviewers.docx [file pone.0257381.s005.docx]

Dear Ms Frey,

Please find attached our responses to the fourth reviewer. We hope that we managed to address in a suitable fashion all the commentaries that have been made for us.

1. I could not see the “overall” recommendations as a separate part of the manuscript.

*In the previous recommendations, you suggested us the following ”Recommendations are suggested to be detailed”. We assumed that the recommendations you are referring to are in regard to the recommendations for improving the system. We modified at that time our recommendations, detailed them and they are presented in the end of our manuscript, where they are easy to be observed. However, we consider that presenting the recommendations in a separate part of the manuscript (as we did with the limitations of our study, according to your suggestion) would contravene to the general structure of an original research paper*.

2. The authors stated that their study was the first study. However, there may be

similar studies and the authors could not have accessed. They should change their sentence.

*At the moment when we submitted the original research article, more than one year ago, our study was the first study which addressed the preparedness phase of the COVID-19 pandemic. At your suggestion, we searched for recent qualitative studies regarding the way in which the healthcare staff experienced the preparedness period, but we did not identify any new studies regarding this topic. However, we changed our statement, adding the fact ”as far as we know” our study is the first study addressing this problem, in order to cover the studies which may have been published, but that are not available on PubMed.*

3. It is still not clear how the authors have connected the “Bridge to heaven” part with

evidence-based medicine.

*Given the fact that this study is a qualitative one in its nature, a sort of subjectivism is inherent to this design. However, we linked evidence-based medicine (if we can name evidence-based medicine the informations which were available at that time - they were mostly statements of different societies) to the title of the theme ”A bridge to heaven”, because, after we immersed into the data obtained from our participants, we considered that this emerged from it. As we tried to illustrate with our quotes, it was clear that for the healthcare staff any piece of trusted information made them feel more secure, gave them courage and energy to fully engage in this fight with the unknown, and together with other measures, we considered that this too is a bridge to heaven, heaven being here a metaphore for their mental well-being, which was absolutely mandatory in those times.*

4. As it is a qualitative research, social scientist’s evaluations should be officially

received before giving the decision.

*As far as we know, the 3^rd^ reviewer, which choosed to make their name fully available for the peer-review process, is an expert in social sciences.*

Note. No need to send the revised manuscript to me, Editorial Office can give the final

decision especially using the social scientist's decision.
